# Innovative Antibiotic Therapies for Carbapenem-Resistant Gram-Negative Bacterial Infections: Clinical Efficacy, Safety, and Comparative Studies

**DOI:** 10.3390/microorganisms13020295

**Published:** 2025-01-29

**Authors:** Majid Eslami, Amirabbas Safaripour, Seyedeh Zahra Banihashemian, Sahar Nikjoo Niaragh, Mohammad Amin Hemmati, Arefeh Shojaeian, Setayesh Fakhariyan, Atiye Rabbani, Valentyn Oksenych

**Affiliations:** 1 Cancer Research Center, Semnan University of Medical Sciences, Semnan 35147-99442, Iran; m.eslami@semums.ac.ir; 2 Department of Bacteriology and Virology, Faculty of Medicine, Semnan University of Medical Sciences, Semnan 35147-99442, Iran; 3 Department of General Surgery, Yasuj University of Medical Sciences, Yasuj 75917-41417, Iran; 4 Student Research Committee, Semnan University of Medical Sciences, Semnan 35147-99442, Iran; 5 Faculty of Medicine, University of Bergen, 5020 Bergen, Norway

**Keywords:** carbapenem, pharmacokinetics, *Acinetobacter baumannii*, *Pseudomonas aeruginosa*, multidrug-resistant

## Abstract

This review provides an overview of recent research and advancements in infection prevention and the treatment of drug-resistant bacterial diseases. Cefiderocol, a novel siderophore cephalosporin, has demonstrated effectiveness against carbapenem-resistant bacteria such as *Pseudomonas aeruginosa* and *Acinetobacter baumannii*. Clinical trials, including APEKS-NP and CREDIBLE-CR, affirm its efficacy for hospital-acquired pneumonia (HAP) but highlight concerns over increased mortality due to severe renal complications. Cefiderocol has shown superior outcomes in complicated urinary tract infections (cUTI) compared to imipenem–cilastatin. A comparison of colistin monotherapy versus combination therapy with meropenem for carbapenem-resistant infections revealed no significant improvement in clinical outcomes with combination therapy but noted delays in resistance development. Colistin–rifampicin combination therapy showed potential benefits for colistin-resistant *Acinetobacter baumannii*, although results were not statistically significant. SPR206, a polymyxin derivative, and durlobactam, a β-lactamase inhibitor, show promise in addressing these resistant strains, with durlobactam demonstrating efficacy in combination with sulbactam and imipenem–cilastatin. Additional studies investigated antibiotic strategies for resistant infections, including cefoperazone–sulbactam versus combination therapy with tigecycline, and examined infection-prevention strategies in surgical settings, comparing chlorhexidine–alcohol and povidone–iodine. This research highlights the importance of optimizing treatment regimens and infection-control measures across various healthcare settings, including neonatology and surgical care.

## 1. Cefiderocol and Novel Antibiotic Treatments for Gram-Negative Bacteria

Cefiderocol, a novel siderophore cephalosporin, has been developed to address infections caused by carbapenem-resistant bacteria, particularly *Pseudomonas aeruginosa* and *Acinetobacter baumannii*. Its efficacy has been validated in various studies, including the APEKS-NP trial, which demonstrated its effectiveness in treating hospital-acquired pneumonia [1]. The CREDIBLE-CR trial was designed to evaluate the efficacy of cefiderocol in infections resistant to carbapenems. The study enrolled patients aged 18 and older who were diagnosed with hospital-acquired pneumonia, ventilator-associated pneumonia, healthcare-associated pneumonia, bloodstream infections, sepsis, or complicated urinary tract infections [2]. Patients were also required to have evidence of carbapenem resistance, such as failure of carbapenem therapy after at least two days, resistance rates exceeding 90% in antibiogram, or confirmation of infections caused by *Stenotrophomonas maltophilia*, a pathogen intrinsically resistant to carbapenems. Participants were randomized based on their infection type and geographical location [3].

Between 7 September 2016, and 22 April 2019, 152 patients were enrolled in the study, with the most common carbapenem-resistant pathogens identified as *Acinetobacter baumannii*, *Klebsiella pneumoniae*, and *Pseudomonas aeruginosa*. Of these, 101 patients were randomly assigned to cefiderocol treatment, while 51 received the best available therapy. The study showed that cefiderocol was as effective as the best available treatments for carbapenem-resistant gram-negative bacteria and performed particularly well against infections caused by metallo-β-lactamase-producing bacteria. However, higher mortality rates were observed among patients treated with cefiderocol, especially in those with *Acinetobacter* infections, likely due to severe kidney complications, as well as among patients with bloodstream infections and sepsis. Almost all patients experienced at least one adverse event, such as diarrhea, fever, septic shock, or vomiting. Liver-related issues, including elevated enzymes and coagulation problems, were more common in the cefiderocol group. Despite its iron-chelating properties, cefiderocol did not cause any direct issues related to iron metabolism or coagulation [4].

Out of 101 patients treated with cefiderocol, 34 died, compared to 9 out of 50 patients treated with the best available therapy. None of the deaths in the cefiderocol group were directly linked to the drug, while one death in the other group was associated with acute kidney injury. Although the mortality rate was higher in the cefiderocol group, there was no clear evidence that the drug caused toxicity. Most deaths in the cefiderocol group occurred within the first three days or after the 29th day of treatment, with similar mortality rates between both groups during days 4 to 28. Carbapenemase-producing *Acinetobacter baumannii*, *Klebsiella pneumoniae*, and *Pseudomonas aeruginosa* remain highly resistant to treatment. Cefiderocol, a novel siderophore cephalosporin, has shown effectiveness against both carbapenem-sensitive and carbapenem-resistant Gram-negative bacteria, though it is not effective against Gram-positive or anaerobic pathogens. The pharmacokinetics of cefiderocol, as demonstrated in the APEKS-NP trial, display linearity, with the drug being excreted entirely by the kidneys. The APEKS-NP trial aimed to evaluate the effectiveness of cefiderocol compared to prolonged high-dose meropenem infusion in patients with hospital-acquired pneumonia (HAP), ventilator-associated pneumonia (VAP), and healthcare-associated pneumonia (HCAP) caused by Gram-negative bacteria [4]. The trial was conducted across 76 hospitals in 17 countries, spanning Asia, Europe, and the Americas. Adults aged 18 years and older with Gram-negative HAP, VAP, or HCAP participated in the study. Participants were randomly assigned to two groups based on infection type and severity. One group received 2 g of cefiderocol via intravenous infusion in 100 mL of saline over three hours every eight hours. In patients with creatinine clearance exceeding 120 mL/min, cefiderocol was administered every six hours. The other group received 2 g of meropenem in 100 mL of saline over three hours every eight hours. The treatment duration for both drugs ranged from 7 to 14 days, with possible extension to 21 days. Additionally, all patients were given 600 mg of linezolid via intravenous infusion every 12 h for five days to prevent Gram-positive bacterial infections, including methicillin-resistant *Staphylococcus aureus* (MRSA), in both groups [5].

From 23 October 2017, to 14 April 2019, a total of 300 patients were selected, with 148 receiving cefiderocol and 152 receiving meropenem. Due to various issues (e.g., three patients with Gram-positive pneumonia and two meropenem-treated patients not receiving the assigned treatment), 292 patients ultimately completed the study. The most common pathogens in these pneumonia cases were *Klebsiella pneumoniae*, *Pseudomonas aeruginosa*, and *Acinetobacter baumannii*. Adverse events were observed in 130 out of 148 patients treated with cefiderocol and in 129 out of 150 patients treated with meropenem. Notable adverse events included urinary tract infections in the cefiderocol group and hypokalemia in the meropenem group, along with gastrointestinal issues such as diarrhea and constipation. Anemia- and liver-related side effects were not observed. A total of 38 out of 142 patients in the cefiderocol group and 34 out of 146 patients in the meropenem group had passed away by the time the study was over. Cefiderocol was efficacious and well-tolerated in critically sick patients whose hospital-acquired pneumonia did not respond to meropenem monotherapy, even though it did not significantly reduce mortality when compared to extended high-dose meropenem infusion. For individuals with HAP brought on by multidrug-resistant (MDR) Gram-negative infections, cefiderocol may therefore be a good course of treatment [5].

Another related study was conducted across 67 hospitals in 15 countries, involving 495 adult patients aged 18 years and older with complicated urinary tract infections (cUTI) with or without acute uncomplicated pyelonephritis. After screening, 448 patients were selected. These patients were randomly assigned to two groups between 5 February 2015, and 16 August 2016. A total of 300 patients received cefiderocol, while 148 patients were treated with imipenem–cilastatin. The cefiderocol group received 2 g of the drug via intravenous infusion, while the imipenem–cilastatin group received 1 g of the combination drug via intravenous infusion over one hour every eight hours, for a treatment duration of 7 to 14 days [6]. The most common pathogens in cUTI cases were *Escherichia coli* and *Klebsiella pneumoniae*. Patients were evaluated on day four (the final day of treatment), seven days after treatment completion for clinical improvement, and fourteen days after treatment completion for follow-up. Safety was assessed by monitoring vital signs, side effects, body temperature, urinalysis, blood tests, and electrocardiography. The microbiological response in patients treated with cefiderocol was superior to that observed in the imipenem–cilastatin group, though clinical improvement was similar in both groups. Common side effects included gastrointestinal disturbances such as diarrhea, constipation, nausea, vomiting, and abdominal pain. Other side effects included hypertension, headache, hypokalemia, renal cysts, insomnia, upper abdominal pain, injection site erythema, heart failure, vaginal infections, and *Clostridium difficile* colitis, the latter being more prevalent in the imipenem–cilastatin group. Severe side effects, including *C. difficile* colitis, occurred in one patient treated with cefiderocol and two patients in the imipenem–cilastatin group. According to the researchers, *C. difficile* colitis was attributed to treatment in the cefiderocol patient and one of the imipenem–cilastatin patients. *C. difficile* colitis in the patients mentioned may not have been directly caused by the treatment itself but rather could have been exacerbated by the disruption of the gut microbiota due to the use of broad-spectrum antibiotics. The treatments, specifically cefiderocol and imipenem–cilastatin, are known to significantly alter the gut microbial environment, which can create an imbalance favoring the overgrowth of *C. difficile* if it is already present in the gut. This bacterium can exist in a dormant state in some patients, and antibiotic treatment may suppress competing bacteria, enabling it to proliferate and produce toxins, leading to colitis. In conclusion, cefiderocol demonstrated better pathogen-eradication from a microbiological perspective compared to imipenem–cilastatin and was well-tolerated by patients. Additionally, it caused fewer side effects than imipenem–cilastatin [6,7,8,9].

## 2. Comparative Studies of Colistin-Based Therapies for Carbapenem-Resistant Bacterial Infections

Adults with ventilator-associated pneumonia, pneumonia acquired in hospitals, and urosepsis due to Gram-negative bacteria resistant to carbapenem were the subjects of another study. The infecting bacteria in this study had to be resistant to all antibiotics, with the exception of colistin, aminoglycosides, sulbactam, tetracyclines, tigecycline, and cotrimoxazole, in order to be included in the study [10,11]. The subjects were split into two groups at random: one group had intravenous colistin alone, and the other group received intravenous colistin plus meropenem. The dosage of colistin consisted of a 9-million-unit loading dose that was followed by 4.5 million units every 12 h. Meropenem was given as a long-term infusion of 2 g over 3 h, three times a day (every 8 h), to the combination group. Seven days after the initiation of treatment, patient samples were taken to assess effectiveness. In patients with bacteremia and persistent fever, blood cultures were performed every 48h. Fourteen days after the treatment began, patients were evaluated for clinical success or failure. Treatment success was defined by survival, stable hemodynamics, recovery or stabilization of organ failure, stabilization or improvement of partial arterial oxygen pressure to fraction of inspired oxygen (PaO_2_/FiO_2_) in pneumonia patients, and microbiological clearance in bacteremia patients. If any of these criteria were not met, the outcome was classified as clinical failure [12].

Between 1 October 2013, and 31 December 2016, a total of 406 patients were enrolled in the study and randomly assigned to either the colistin monotherapy group (198 patients) or the colistin–meropenem combination group (208 patients). Most patients were treated for pneumonia and bacteremia, primarily caused by *Acinetobacter baumannii*, thus, the results predominantly reflect outcomes for this pathogen. One benefit of the combination therapy was the observed delay in the development of resistance in gram-negative bacteria compared to monotherapy with colistin. However, the study found no significant difference in clinical outcomes between the two groups. The combination therapy did increase the incidence of diarrhea but reduced mild renal failure. Nonetheless, the combination treatment was not recommended solely for renal protection. Clinical failure occurred in 308 out of 406 patients by the 14th day, with no significant differences between the two treatment groups. One limitation of the study was the lack of real-time drug-concentration monitoring to guide therapy, though colistin was administered at doses sufficient to achieve the necessary plasma concentrations for bacterial susceptibility in most patients. By day 14, 134 patients had died [12].

The study also explored the effects of colistin and colistin–rifampicin combination therapy in patients over 18 years old with pneumonia caused by colistin-resistant *Acinetobacter baumannii*. Participants who had allergic reactions to rifampicin, had received colistin or rifampicin within the last 15 days, or had experienced severe drug-related side effects were excluded from the study. From 20 July 2016 to 21 June 2018, 18 patients were enrolled, but only 9 continued due to exclusion criteria, and 1 additional patient left the study due to a hospital transfer, leaving 8 patients for analysis. These patients were randomly assigned to receive either colistin monotherapy or colistin–rifampicin combination therapy. Those in the colistin monotherapy group received 100 mg of colistin in 100 mL of normal saline every 8 h, while those in the combination group received the same colistin regimen plus 600 mg of oral rifampicin daily. The treatment duration ranged from 7 to 28 days, and no serious complications were reported in either group [13].

The results indicated that the colistin–rifampicin combination therapy had better outcomes than colistin monotherapy in eradicating *Acinetobacter baumannii*, although the difference was not statistically significant. Another benefit of combination therapy was the prevention of resistance development against colistin. *Acinetobacter baumannii* is a highly resistant pathogen that frequently causes infections in hospital settings, and when resistant to carbapenems, colistin is often the only remaining treatment option. There are currently few antibiotics in development that effectively combat this bacterium, and combination therapy, while used in practice, has limited evidence supporting its effectiveness [13,14].

The mortality rates of patients infected with carbapenem-resistant *Acinetobacter baumannii* were examined in a study, and the results of monotherapy and combination therapy with colistin did not significantly differ. Microbiological testing for antibiotic susceptibility is essential since colistin-resistant strains of this bacterium are currently infecting an increasing number of patients. However, there may be differences in the preliminary susceptibility findings due to the limitations of standard testing methodologies. Patients who were first thought to be susceptible to colistin were occasionally discovered to be resistant. This gave researchers the chance to investigate the connection between colistin resistance, antibiotic therapy, and patient mortality [15,16].

In a randomized, controlled trial on carbapenem-resistant Gram-negative bacterial infections, patients were treated with either colistin monotherapy or a combination of colistin and meropenem. A secondary analysis focused on patients with *A. baumannii* infections, originally identified as colistin-susceptible (CoS), and compared them to those later confirmed to be colistin-resistant (CoR) through broth microdilution testing. The primary outcome measured was 28-day mortality. Data from 266 patients infected with either CoS or CoR strains were analyzed, with the majority of infections caused by CoS isolates. Interestingly, patients with CoR infections tended to have better functional statuses prior to infection and were less likely to require mechanical ventilation. The analysis revealed a lower 28-day mortality rate in patients with CoR infections compared to CoS infections, though the difference was not statistically significant. After adjusting for confounding factors, patients with CoR isolates had a significantly lower mortality rate. However, patients receiving colistin–meropenem combination therapy for CoR infections experienced higher mortality rates compared to those treated with colistin monotherapy [17].

## 3. Pharmacokinetics and Safety of Durlobactam, SPR206, and Related Antibiotics

The rise of carbapenem-resistant *Acinetobacter baumannii*, *Enterobacterales*, and MDR *Pseudomonas aeruginosa* presents significant public health challenges due to their role in causing fatal infections. Current research suggests that SPR206, a new polymyxin derivative with potent in vitro and in vivo activity, could offer a promising alternative to traditional antibiotics such as polymyxin, colistin, meropenem, and aminoglycosides in combating serious MDR infections [18]. In a double-blind, placebo-controlled, first-in-human study, SPR206 was administered as a single intravenous (IV) dose during the Single Ascending Dose (SAD) phase, and in the Multiple Ascending Dose (MAD) phase, it was given intravenously every eight hours (q8h) for 7 to 14 days. Results indicated that SPR206 was well-tolerated, with minimal adverse effects. Mild paresthesia was observed at higher doses, but there were no significant effects on renal, cardiac, or hepatic function. Furthermore, SPR206 displayed a dose-proportional pharmacokinetic (PK) profile, achieving steady-state by day two. Its reduced nephrotoxicity compared to other polymyxins suggests it could be a viable therapeutic option [18].

Durlobactam, a novel drug targeting antibiotic resistance, functions by inhibiting beta-lactamase enzymes, which are responsible for bacterial resistance to beta-lactam antibiotics. Durlobactam has shown efficacy against a variety of beta-lactamases, including those in *Acinetobacter baumannii*, a frequent cause of hospital-acquired infections. Sulbactam, another beta-lactamase inhibitor, has been used to treat *A. baumannii* infections but faces limitations due to emerging resistance. A study evaluating durlobactam’s safety and pharmacokinetics was conducted in healthy subjects across four groups: durlobactam alone, durlobactam plus sulbactam, durlobactam plus imipenem–cilastatin (IMI-CIL), and a combination of all three drugs. The results showed that durlobactam was well-tolerated across all doses, with mild side effects like somnolence and nausea, which resolved without intervention. Pharmacokinetic analysis revealed no significant drug–drug interactions, suggesting that durlobactam can be safely combined with other antibiotics [19].

Durlobactam (formerly ETX2514), a novel diazabicyclooctane β-lactamase inhibitor, has shown promise in overcoming resistance caused by class A, C, and D β-lactamases, particularly in *Acinetobacter baumannii* infections. When combined with sulbactam, it demonstrates significant efficacy against carbapenem- and colistin-resistant strains, addressing a critical need for new treatments for MDR infections. Clinical studies have shown that durlobactam is well-tolerated, with only mild, transient side effects such as somnolence and nausea. Its pharmacokinetic profile reveals no significant drug–drug interactions, supporting its use as an adjunct to β-lactam antibiotics like sulbactam and imipenem–cilastatin [20]. Durlobactam, unlike macrolides, does not pose a risk of QT prolongation and is intended specifically as an adjunctive therapy to β-lactam drugs rather than as a standalone treatment. By focusing on its role in enhancing the efficacy of penicillin-type antibiotics, durlobactam represents a significant advancement in combating MDR bacterial infections, especially in the context of limited current options. A double-blind, three-way crossover study on healthy individuals randomized them to receive one of three treatments: a single 3 h IV infusion of durlobactam (4 g, supratherapeutic dose), placebo, or placebo with an oral dose of moxifloxacin (400 mg). Carbapenems, such as imipenem, are broad-spectrum antibiotics effective against severe infections caused by Gram-positive and Gram-negative bacteria and are commonly used to treat urinary, respiratory, abdominal, and polymicrobial infections. In pediatric patients with hematological malignancies, an imipenem pharmacokinetic study revealed that approximately 70% of the drug is renally excreted. Time above the MIC was identified as a key indicator of therapeutic efficacy. Although adult studies showed increased clearance correlated with creatinine clearance (CLCR), pediatric analyses indicated that body weight, age, and renal function were the primary factors influencing imipenem pharmacokinetics. The increasing resistance of *Pseudomonas aeruginosa* and *A. baumannii* to imipenem has rendered the standard 25 mg/kg q6h dosing regimen ineffective against resistant strains, necessitating alternative therapeutic strategies [21].

**Figure 1:**
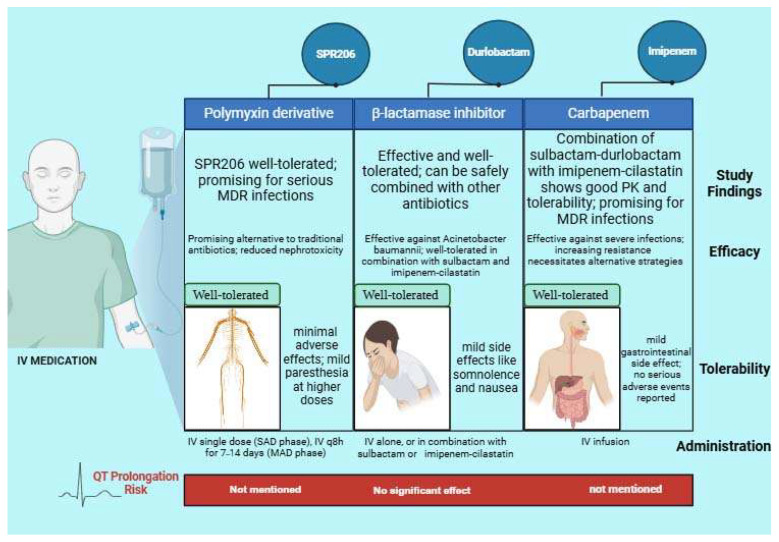
Efficacy, tolerability, and administration of durlobactam, SPR206, and imipenem.

Recent findings confirm that durlobactam’s PK profile remains consistent with previous studies and does not significantly affect the QT interval, eliminating concerns about proarrhythmic effects. The combination of durlobactam and sulbactam demonstrated safety with low proarrhythmic risk, making it a potential candidate for treating infections caused by MDR bacteria. [20]. In a study of 32 adults hospitalized with cUTIs, including acute pyelonephritis, participants received intravenous sulbactam–durlobactam (1.5 g) and imipenem–cilastatin (500 mg) every six hours for 4 to 14 days. The peak concentration of sulbactam–durlobactam in plasma was 112 µg/mL, and the area under the curve (AUC) was 553 µg·h/mL. For imipenem, the mean maximum concentration was 25 µg/mL, with an AUC of 79 µg·h/mL. The elimination half-lives were 2.2 h for sulbactam–durlobactam and 1.6 h for imipenem. The treatment was well-tolerated, with mild gastrointestinal side effects like diarrhea, nausea, and vomiting, and no serious adverse events or deaths reported. In conclusion, imipenem–cilastatin combined with sulbactam–durlobactam showed good pharmacokinetics and was well-tolerated in cUTI patients. This combination holds promise as a therapeutic option for infections caused by MDR Gram-negative bacteria. The findings underscore the potential of β-lactamase inhibitors like sulbactam–durlobactam, used alongside carbapenems, to address antibiotic-resistant infections. These insights contribute valuable pharmacokinetic data, laying the foundation for future research and development of more effective treatment regimens for MDR bacterial infections [22]. (Figure 1).

## 4. Effectiveness of Multi-Drug Regimens for Resistant *Acinetobacter baumannii* Infections

Another study determined which combination of cefoperazone–sulbactam and tigecycline is more effective at treating lung infections caused by MDR *Acinetobacter baumannii* [23]. Patients over 18 years old who had been hospitalized for more than 48 h with sputum culture and imaging results confirming MDR *Acinetobacter baumannii* pneumonia were included in the study. Patients were excluded if they had conditions such as pulmonary tuberculosis, immunological disorders, mental health issues impairing communication, recent antibiotic use, drug allergies, pregnancy or breastfeeding, or were unable to complete the treatment [24,25,26]. Patients were randomly assigned to two groups using a random number table, with 57 participants in each group. Group A received cefoperazone–sulbactam sodium alone, while Group B was administered a combination of cefoperazone–sulbactam and tigecycline continuously for 14 days. During the treatment period, vital signs were closely monitored, and fluid-electrolyte and acid-base balances were carefully managed. Blood samples were collected both before and after the 14-day treatment to evaluate levels of procalcitonin (PCT), C-reactive protein (CRP), tumor necrosis factor-alpha (TNF-α), and interleukin-6 (IL-6). Additionally, the APACHE II score was used to assess patient prognosis [27]. Among the 114 patients, 60 (52.63%) were male and 54 (47.37%) were female, with ages ranging from 26 to 77 years (mean age: 62.18 ± 4.25 years). Underlying conditions included coronary heart disease in 24 patients (21.05%) and diabetes mellitus in 13 patients (11.40%). Prior to treatment, no significant differences were observed between Group A and Group B in the serum levels of PCT, CRP, TNF-α, and IL-6 (*p*-values: 0.889, 0.426, 0.794, and 0.946, respectively). After 14 days of treatment, the levels of these markers in Group B were significantly lower compared to Group A (all *p*-values < 0.001, see Table 1), and the APACHE II scores in Group B were also significantly lower than those in Group A post-treatment [27].

*Acinetobacter baumannii* releases endotoxins and LPS during infection, which can stimulate the release of inflammatory factors from mononuclear macrophages and neutrophils, potentially leading to systemic inflammatory response syndrome. Clinically, CRP, TNF-α, and IL-6 are utilized to assess inflammatory responses, with CRP produced by hepatocytes in response to inflammation and TNF-α and IL-6 playing roles in immune cell activation and serving as key indicators of bacterial infection. PCT is also a critical marker for infection severity [28].

The researchers demonstrated that the combination of cefoperazone–sulbactam and tigecycline was more effective than cefoperazone–sulbactam alone in reducing serum inflammatory markers such as procalcitonin (PCT), C-reactive protein (CRP), TNF-α, and IL-6. They reported significantly lower APACHE II scores in patients treated with the combination therapy, suggesting improved clinical outcomes and a better prognosis. The authors highlighted the necessity for further studies to evaluate bacteriological clearance rates and validate the long-term effectiveness of this treatment in managing *Acinetobacter baumannii* pulmonary infections [29].

Ventilator-associated pneumonia (VAP) is a serious lower respiratory tract infection with increasing mortality rates and treatment costs [30]. In critically ill Iranian patients, the incidence of VAP ranges from 20% to 30%, with *Acinetobacter baumannii* being a significant causative agent. The incidence of VAP due to MDR *A. baumannii* ranges from 1.9% to 18.3% per 1000 ventilator days [31]. Currently, *Acinetobacter* spp. exhibit resistance to a broad spectrum of antibiotics, including carbapenems, with approximately 63% of isolates being MDR. Regarding antibiotic options, colistin is a potential treatment for carbapenem-resistant *A. baumannii*, though its resistance rates vary globally from 0% to 40.6%. Sulbactam, an irreversible β-lactamase inhibitor, shows significant activity against *A. baumannii* [32]. For carbapenem-resistant isolates, the synergistic effects of combining carbapenems with other antimicrobial agents can be explored. Patient-specific factors also influence the effectiveness of antibiotic regimens. There was no discernible difference in the clinical recovery rates of 47 VAP patients treated with meropenem/colistin or meropenem/ampicillin–sulbactam in a randomized clinical research trial. The patients’ death rates during ICU admission were 75% and 69.6%, respectively. These results suggest that ampicillin–sulbactam could be utilized as a substitute for colistin in the treatment of VAP brought on by *A. baumannii* that is resistant to carbapenems. Since sulbactam is unavailable in Iran, ampicillin–sulbactam is utilized in its stead. Creating a successful antibiotic regimen is still essential for treating these infections [31,33].

## 5. Antibiotic Treatment and Outcomes for Carbapenem-Resistant Gram-Negative Bacterial Infections

Carbapenem-resistant Gram-negative bacteria (CRGNB) create significant challenges for healthcare providers due to limited treatment options and high mortality rates. Healthcare professionals frequently use empirical antibiotic therapy to manage severe infections caused by CRGNB, but its impact on patient outcomes remains poorly understood. To address this gap, a prospective observational study was conducted to evaluate the relationship between empirical antibiotic treatment and mortality risk in patients with severe CRGNB infections. This study included 107 patients from three hospitals in China who were diagnosed with CRGNB infections between January 2016 and December 2017. Participants were categorized into two groups based on the receipt of empirical antibiotic treatment. The findings revealed an overall mortality rate of 46.7% within the study cohort. Patients who received empirical antibiotic treatment exhibited a significantly lower mortality rate (36.4%) compared to those who did not receive such treatment (66.7%). Multivariate logistic regression analysis further indicated that empirical antibiotic therapy was independently associated with a decreased risk of mortality [34].

Additionally, this study demonstrated that monotherapy with carbapenems was linked to a higher mortality rate compared to combination therapy with other antibiotics, suggesting that combination therapy may offer more effective management of severe CRGNB infections. Despite its limitations—such as a small sample size and retrospective design—this study suggests that empirical antibiotic treatment may enhance recovery in patients with severe CRGNB infections. Future research is essential to validate these findings and determine optimal treatment strategies for these complex infections. In a related context, this prospective study also evaluates the use of empirical antibiotic treatment based on in vitro studies suggesting that combinations of polymyxins (e.g., colistin) and carbapenems might improve effectiveness and prevent resistance in CRGNB infections. However, two meta-analyses have reported that such combinations do not reduce mortality. This study compares in vitro synergy results with clinical outcomes to assess the relevance of these findings in improving patient care [34].

A total of 171 patients with CRGNB infections, including isolates of *Acinetobacter baumannii*, *Enterobacteriaceae*, and *Pseudomonas aeruginosa*, were included in this study, which found that combination therapy did not significantly impact primary outcomes, such as 14-day clinical failure, nor did it affect secondary outcomes like 14-day and 18-day mortality rates or microbiological failure. These findings are consistent with the AIDA trial, a multicenter randomized controlled trial (RCT) involving 406 patients, which also showed that adding meropenem to colistin did not provide additional benefits compared to colistin monotherapy [35]. This study serves as a secondary analysis of the AIDA trial, assessing minimal and fractional inhibitory concentrations (MIC and FIC) to determine synergism (∑FIC_min ≤ 0.5), antagonism (∑FIC_max > 4), or additivity. This analysis showed that *A. baumannii* exhibited 53% synergism, while *Enterobacteriaceae* demonstrated 43% antagonism. The synergism group primarily included patients with pneumonia, while the antagonism group predominantly had bacteremia. In secondary analyses of the AIDA trial, patients with strains exhibiting drug synergism did not show clinical benefits [35]. Bremmer et al. also tested doripenem–colistin combinations in six patients with extensively drug-resistant (XDR) *A. baumannii*, finding in vitro synergy in two cases but clinical failure overall [36].

Several factors might explain the discrepancy between in vitro results and clinical outcomes:

1. Differences in Drug Concentrations: In vivo drug concentrations often differ significantly from those tested in vitro. The concentrations required for efficacy in a controlled laboratory environment may not accurately reflect the levels achievable within the human body, where pharmacokinetics, such as absorption, distribution, metabolism, and elimination, influence drug availability at the infection site.
2. Host–Pathogen Interactions: In vitro studies typically fail to account for the intricate interactions between the host’s immune system and the pathogen. In a living organism, the immune response plays a key role in modulating the effect of antimicrobial therapy, including immune cell recruitment, inflammation, and cytokine production. These factors are not replicated in laboratory settings, where pathogens are isolated from the complexities of host defense mechanisms.
3. Testing Methods: The methods used for synergy testing in vitro are often limited in scope, as they generally rely on a single testing approach. This may not fully capture the variability and dynamic interactions that occur between bacterial pathogens and antibiotics in vivo. Different methods, such as time-kill assays or animal models, might yield more accurate predictions of clinical efficacy, but they are often not employed in initial in vitro studies.
4. Checkerboard Assay Limitations: The checkerboard assay, commonly used to assess drug synergy, primarily measures bacteriostatic activity, which may not be sufficient to achieve a clinical cure. Bactericidal activity, necessary for eradicating infections, is not always adequately demonstrated in this assay. Furthermore, the assay does not mimic the changing concentrations of drugs that occur in the body over time, which may impact therapeutic outcomes. These limitations underscore the complexity of translating in vitro findings into clinical practice. While in vitro studies can provide valuable insights into potential treatment strategies, they cannot fully replicate the complexities of human infection. Therefore, further research, including clinical trials and advanced in vivo models, is needed to optimize treatment regimens for CRGNB infections and improve patient outcomes [37,38].

## 6. Infection Control and Prevention in Surgical and Hospital Settings

The aim of this study was to compare the efficacy of chlorhexidine–alcohol versus povidone–iodine as skin antiseptics in preventing surgical site infections (SSI) following cesarean delivery (CD). This prospective pilot randomized controlled trial included 300 participants, who were divided into two groups:

- Group A: Received chlorhexidine–alcohol (2% chlorhexidine–alcohol)
- Group B: Received povidone–iodine (10% povidone–iodine)

Participants included all pregnant women scheduled for either emergency or elective cesarean deliveries, regardless of gestational age. Exclusion criteria encompassed individuals with a history of allergy to either antiseptic, any adjacent skin infections, severe anemia (hemoglobin < 7 g/dL), fever ≥ 38 °C on two or more occasions within one week prior to CD, signs of chorioamnionitis, use of immunosuppressants, heart disease, uncontrolled diabetes mellitus, or HIV infection. All participants received prophylactic broad-spectrum antibiotics (cefazolin 2g IV) before skin incision. Swabs were taken from the surgical site at three time points: before applying the antiseptic (Swab 1), after applying the antiseptic (Swab 2), and from patients who developed SSI (Swab 3). The primary outcome was the rate of SSI, and secondary outcomes included the growth of organisms on the swabs. Results showed an overall SSI rate of 7%. In Group A, the SSI rate was 5.4%, whereas in Group B, it was 8.6%, with no statistically significant difference between the groups. For secondary outcomes, Swab 1 revealed growth of *Enterococcus faecalis*, *E. coli*, and *Pseudomonas aeruginosa*. No organisms were found in Swab 2. In Swab 3, *E. coli* was identified in 2 patients from Group A, while *Klebsiella pneumoniae* and *Acinetobacter baumannii* were found in Group B. These findings align with several other studies [39,40].

A systematic review and meta-analysis by Noorani et al. reported an SSI rate of 6.1% in the chlorhexidine–alcohol group compared to 9.8% in the povidone–iodine group. Darouiche et al. found a SSI rate of 9.5% with chlorhexidine versus 16.1% with povidone–iodine [41]. Ngai et al. observed similar SSI rates of 4.5% in the chlorhexidine–alcohol group and 4.6% in the povidone–iodine group. Additionally, the CAPICA trial indicated comparable SSI rates between the two antiseptics. The study also assessed the impact of a four-probiotic regimen on SSI prevention in patients with traumatic brain injuries undergoing mechanical ventilation, using data from the ProVAP clinical trial. Patients were randomized to receive either a probiotic formula (containing *Lactobacillus acidophilus* LA-5, *Lactiplantibacillus plantarum* UBLP-40, *Bifidobacterium animalis* subsp. *lactis* BB-12, and *Saccharomyces boulardii* Unique-28) or a placebo containing glucose polymer. The analysis focused on surgical patients within the trial. The results indicated that 46.0% of operations in the placebo group and 24.5% in the probiotic group resulted in infections, with the majority related to osteosynthesis procedures. Pathogens frequently identified included *Staphylococcus aureus* and *Acinetobacter baumannii*. These findings suggest a potential protective effect of probiotics against SSIs. Overall, this study supports the use of probiotics as a preventative measure to reduce SSI incidence in severely traumatized patients, including those with traumatic brain injuries. The four-probiotic regimen appears safe and effective, potentially aiding in microbial dysbiosis following acute trauma and surgical stress. Maintaining or restoring intestinal flora pre- and post-operatively is crucial for patient recovery, given its role in overall health and immune function [42].

Two groups were compared in a research trial on patients having pancreaticoduodenectomy: the goal-directed fluid therapy (GDFT) group and the liberal fluid infusion (LFI) group. Fecal samples were analyzed pre- and post-operatively. Alpha and beta diversity analyses showed significant differences in intestinal flora between the groups. The GDFT group demonstrated better reconstruction of intestinal flora post-surgery, with notable differences involving *Prevotella*, *Roseburia*, and *Lachnospiraceae incertae sedis*. The LFI group showed significant effects from *Enterococcus*, *Pseudomonas aeruginosa*, and *Acinetobacter baumannii*. The GDFT group had more beneficial anaerobic glycolysis metabolism, while the LFI group had more opportunistic pathogens [43].

## 7. Pharmacokinetics, Dosage, and Clinical Outcomes of Antibiotics in Specific Populations

Carbapenems are broad-spectrum antibiotics effective against a variety of severe infections caused by both Gram-positive and Gram-negative bacteria. These drugs are commonly used to treat infections in the urinary, respiratory, and abdominal systems, as well as other polymicrobial infections [44]. Imipenem, a carbapenem, has been studied in pediatric populations with hematological malignancies. Analysis of pharmacokinetic data indicated that 70% of imipenem is excreted renally. For assessing therapeutic efficacy, the duration during which the free drug concentration exceeds the MIC is a crucial parameter [21]. Although studies in adults have shown that clearance increases with CLCR, no covariates, including CLCR, significantly impacted the pharmacokinetic parameters of imipenem. Pharmacokinetic data for children and infants differ from those for adults, with current weight, age, and renal clearance being significant factors in imipenem pharmacokinetics in pediatric patients [45,46].

Imipenem has shown good efficacy against pathogens with low resistance rates, such as *E. coli* and *K. pneumoniae*. However, *Pseudomonas aeruginosa* and *Acinetobacter baumannii* have increasingly developed resistance to imipenem [47,48]. The standard dosing regimen of 25 mg/kg every 6 h is insufficient against these resistant pathogens. Therefore, higher doses or alternative antimicrobial therapies should be considered for treating infections caused by *P. aeruginosa* and *A. baumannii* in children. Lower respiratory tract infections (LRTIs) such as pneumonia are often treated with ciprofloxacin, a broad-spectrum antibiotic effective against Gram-negative bacteria like *P. aeruginosa*, which is common in elderly patients. Ciprofloxacin is a concentration-dependent antibiotic, and inappropriate dosing can lead to poor clinical outcomes. Due to rising drug resistance, finding effective antibiotics remains challenging [49].

In a study involving 43 elderly patients with LRTIs, 33 patients with confirmed Gram-negative infections (based on tracheal aspirate or sputum culture) were analyzed. Exclusion criteria included HIV positivity, tuberculosis, or other infections. Ciprofloxacin was administered in combination with beta-lactams, with doses ranging from 200–400 mg every 12 h, based on infection severity and renal function. Blood samples were collected to evaluate clinical success and bacteriological effects (complete pathogen eradication). Isolated pathogens included *P. aeruginosa* (17 patients), *A. baumannii* (14 patients), and *K. pneumoniae* (2 patients). Eight patients achieved clinical success, with significantly higher AUC/MIC and Cmax/MIC ratios compared to the clinical failure group. The thresholds for AUC/MIC and Cmax/MIC were 40.9 and 3.7, respectively, but most patients did not reach these target values. Among patients with clinical success, seven also experienced bacteriological success. The synergistic effect of ciprofloxacin and beta-lactams may explain the clinical success observed, even when target AUC/MIC values were not met [50].

The emergence of antibiotic-resistant bacteria has prompted the development of new antimicrobial agents, such as ETX2514SUL, a combination of the β-lactamase inhibitor sulbactam and the novel cephalosporin ETX2514. This research aimed to evaluate the pharmacokinetics of ETX2514SUL in healthy adults. Twelve participants received a single 2 h intravenous dose of the drug combination, with blood and bronchoalveolar lavage (BAL) fluid samples collected at various times. The maximum plasma concentrations of ETX2514 and sulbactam were 44.1 µg/mL and 69.8 µg/mL, respectively. The average total drug concentrations were 136 µg·h/mL for ETX2514 and 282 µg·h/mL for sulbactam. The mean ETX2514 concentration in BAL fluid was 1.59 µg/mL, while sulbactam was undetectable in BAL fluid samples [51].

**Figure 2:**
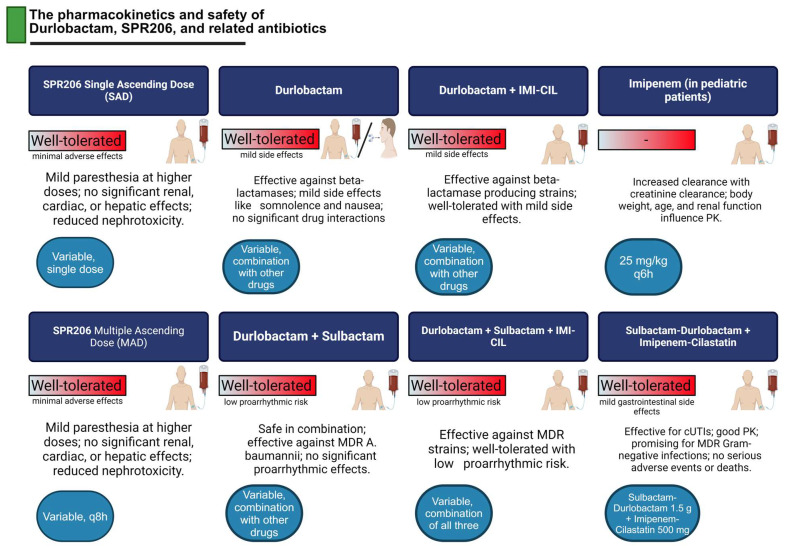
Pharmacokinetics, dosage, and clinical outcomes of durlobactam, SPR206, and related antibiotics.

These findings suggest that ETX2514 and sulbactam are rapidly and efficiently distributed into plasma following intravenous administration. However, the intrapulmonary concentration of ETX2514 was lower than its plasma concentration, and sulbactam was not detected in the BAL fluid. This indicates a need for further research to optimize ETX2514SUL dosing and frequency for treating lung infections caused by MDR bacteria. Overall, this study provides valuable pharmacokinetic data that may inform the development of new treatment strategies for resistant bacterial infections [51]. (Figure 2).

## 8. Bacterial Resistance and Infection Trends in Vulnerable Populations

Antimicrobial resistance (AMR) and fungal sepsis represent critical global health issues, especially among neonates [52]. A study assessed the prevalence of AMR and fungal infections among newborns delivered outside of hospital settings and subsequently admitted to a Neonatal Intensive Care Unit (NICU) in North India. Data from 110 neonates admitted to the NICU from January to December 2017 were analyzed. All participants were born outside the hospital and were transferred from other healthcare facilities. The findings revealed that 45.5% of the neonates had bacterial sepsis, with *Klebsiella pneumoniae* being the predominant pathogen. The study identified a high prevalence of AMR, with over 90% of *Klebsiella pneumoniae* strains resistant to third-generation cephalosporins and carbapenems. Additionally, more than 70% of these strains exhibited resistance to amikacin and gentamicin, commonly used aminoglycoside antibiotics. Fungal sepsis was observed in 12.7% of the neonates, with *Candida* species being the most frequently isolated fungi. Antifungal resistance was also notably high, with over 70% of *Candida* strains resistant to fluconazole, a widely used antifungal agent [53]. The study further indicated that neonates with AMR or fungal sepsis experienced a significantly higher mortality rate compared to those without these conditions, with the highest mortality observed in those with both bacterial and fungal sepsis. These findings underscore the urgent need for improved infection-control strategies and judicious antibiotic use in healthcare settings, particularly in neonatal units. The high prevalence of AMR and antifungal resistance highlights the necessity for appropriate antimicrobial stewardship and targeted therapies to mitigate treatment failures and associated mortality. In conclusion, this study highlights alarming rates of AMR and fungal sepsis among outborn neonates in North India, which are linked to elevated mortality rates [53].

Beta-lactam antibiotics, known for their broad-spectrum activity and safety, are commonly used in clinical settings. However, Gram-negative bacteria that produce extended-spectrum beta-lactamases (ESBLs) pose a significant health threat. This study involved 532 patients with bloodstream, urinary tract, wound, and other bacterial infections. Blood samples from adults, children, and neonates were collected and monitored for up to seven days in cases of negative initial blood cultures. Midstream urine samples were processed to identify significant bacteriuria. All bacterial isolates were tested using the disk diffusion method, with an inhibition zone of <22 mm for ceftazidime or <27 mm for cefotaxime indicating potential ESBL production. Phenotypic confirmation was performed using the double diffusion method, while carbapenemase production was assessed using the Modified Hodge Test (MHT). Of the samples tested, 49.4% were culture-positive, with 70.3% of the pathogens being Gram-negative. The ratio of Gram-negative to Gram-positive isolates was approximately 3:1, with *Klebsiella pneumoniae*, *Escherichia coli*, *Acinetobacter baumannii*, and *Enterobacter aerogenes* being the most frequently isolated Gram-negative bacteria. All Gram-negative bacterial isolates were resistant to ampicillin, and most showed resistance to more than nine antibiotics. Cefoxitin demonstrated superior performance compared to other cephalosporins, exhibiting the lowest resistance rate. Ciprofloxacin and cefoxitin were found to be particularly effective against *Klebsiella pneumoniae* [54]. According to Abe et al., Gram-negative bacteremia induces a higher inflammatory response that can impact clinical outcomes. The study found that 127 isolates produced ESBLs and 24 produced carbapenemases, with 80% of all isolates classified as MDR [55].

Intravitreal injections are a key treatment for diabetic retinopathy and other retinal disorders. This study compared prophylactic methods to reduce conjunctival bacterial load, infection risk, and endophthalmitis. A total of 132 patients with neovascular age-related macular degeneration were randomly assigned to three groups. Samples were collected at admission, four days before injection, and four and eight days after injection, using non-contaminated techniques from the lower eyelid fornix. The control group received only 5% povidone–iodine (PI), known to sterilize the ocular surface and prevent endophthalmitis [56].

Groups 1 and 2 received azithromycin or moxifloxacin, four drops per day, with re-prescription at the same dosage post-injection. A 5% PI affects both Gram-negative and Gram-positive bacteria, viruses, and fungi and can eliminate antibiotic-resistant microbes. Coagulase-negative *Staphylococcus* (CNS) was the most frequently identified microorganism (23.8%), followed by *Acinetobacter* spp. (4.5%). Moxifloxacin demonstrated greater effectiveness than 5% PI, with a synergistic effect observed when combined. *Staphylococcus aureus*, *E. coli*, and *A. baumannii* did not grow in the moxifloxacin group. Newer fluoroquinolones were more effective against Gram-positive organisms. Azithromycin, although more effective than erythromycin against Gram-negative bacteria, showed lower penetration capacity, resulting in continued growth of *Acinetobacter* spp. in the azithromycin group. By the fourth sample collection, no significant differences in bacterial growth were observed among the groups. Both azithromycin and moxifloxacin were ineffective in eradicating all microorganisms before and after intravitreal injection, indicating that 5% PI should be used in conjunction for optimal prophylaxis [56,57].

The normal conjunctival flora, including *Staphylococcus epidermidis*, provides protection against pathogens. Recent studies suggest that topical antibiotics after recurrent injections may lead to antibiotic resistance in conjunctival flora. Deve et al. reported that repeated use of fluoroquinolones and azithromycin alters conjunctival flora and increases *S. epidermidis* populations [58]. Disruption of the oral microbiome balance allows opportunistic pathogens to proliferate, contributing to post-stroke aspiration pneumonia. Chlorhexidine is considered the gold standard for oral hygiene, but the efficacy of tooth brushing remains uncertain. This single-blind randomized controlled trial involved two intervention periods of three months each, with 94 subjects randomly assigned to two groups. One group received conventional oral care using a manual toothbrush, while the other received advanced care with a powered toothbrush and chlorhexidine. No antibiotics were administered except to three patients with urethritis [59,60,61,62].

At the study’s outset, aerobic Gram-negative bacteria (AGNB) were isolated in 47.9% of cases, with *Enterobacteriaceae* being the most prevalent family. *Klebsiella pneumoniae* and *Acinetobacter baumannii* were common, and yeasts, particularly *Candida albicans*, were isolated from 50% of participants. Three months of intervention did not alter the prevalence of these microorganisms or their viable counts. By the end of the trial, only the viable counts of *Staphylococcus aureus* decreased in the advanced oral hygiene care group. Cigarette smokers were more likely to harbor AGNB, as smoking impairs mucosal fibronectin covering and affects gingival blood supply, compromising tissue repair. Patients with yeast presence at the study’s start showed higher yeast prevalence at three and six months. Denture use is an established risk factor for yeast colonization and oral hygiene issues. No cases of aspiration pneumonia were reported during the trial. The prevalence hierarchy of microorganisms at the end of the first three months was: *Klebsiella pneumoniae*, *Acinetobacter* spp., and *Pseudomonas aeruginosa* [60].

## 9. Detailed Analysis of Research Gaps and Contributions

Despite the growing body of research on MDR bacterial infections and the development of novel antibiotics, several critical gaps remain in understanding the full scope of treatment options, particularly for infections caused by carbapenem-resistant pathogens. One major gap is the limited exploration of long-term efficacy and safety of new therapies such as cefiderocol, which, although promising, is associated with significant renal toxicity and mortality concerns. Much of the existing research on cefiderocol, such as the APEKS-NP and CREDIBLE-CR trials, has focused primarily on short-term clinical outcomes, particularly in patients with hospital-acquired pneumonia and complicated urinary tract infections. However, these studies have not adequately addressed the broader patient population and the effects of cefiderocol on patients with comorbidities, those undergoing prolonged treatments, or individuals with a history of renal dysfunction.

Additionally, while cefiderocol has shown superior performance in combating metallo-β-lactamase-producing bacteria, its use in clinical settings has been limited to studies on specific pathogens, particularly *Acinetobacter baumannii*, *Klebsiella pneumoniae*, and *Pseudomonas aeruginosa*. The scope of these studies does not fully account for the diverse array of carbapenem-resistant organisms that may present in different healthcare settings. As a result, the long-term impact of cefiderocol on bacterial resistance, microbiome alterations, and the emergence of secondary infections remains underexplored. Furthermore, there is a lack of comprehensive data on the safety of combining cefiderocol with other antibiotic therapies, particularly in severely ill patients who may be undergoing polypharmacy. This study contributes to addressing these gaps by providing a comprehensive analysis of both the short- and long-term outcomes associated with cefiderocol treatment. It not only examines its efficacy against the most resistant Gram-negative bacteria, but it also delves deeper into the renal complications and mortality risks associated with its use. By comparing cefiderocol to the best available therapies, this research highlights its comparative advantages as well as the potential risks that need to be managed in clinical practice, especially among patients with pre-existing conditions or those in critical care settings. Moreover, the study contributes to understanding the broader applicability of cefiderocol by including a diverse cohort of patients and exploring its effectiveness in various types of carbapenem-resistant infections.

Moreover, this work emphasizes the need for further investigation into the optimization of cefiderocol use in combination with other antibiotics, considering both the potential for enhanced efficacy and the risk of adverse interactions or compounded toxicity. By identifying gaps in the current research on MDR infections and proposing areas for further exploration, this study provides a clearer direction for future clinical trials, including those that focus on patient stratification based on comorbid conditions and treatment history. Generally, while this study builds on the existing evidence regarding cefiderocol’s efficacy and safety, it expands the understanding of its role in combating resistant infections by highlighting critical gaps in current knowledge and suggesting strategies to mitigate the risks associated with its use. This contributes to the ongoing efforts to refine treatment regimens and improve patient outcomes, particularly in the face of rising antibiotic resistance.

## 10. Limitations of Cited Studies and Variability in Patient Outcomes

**Figure 3:**
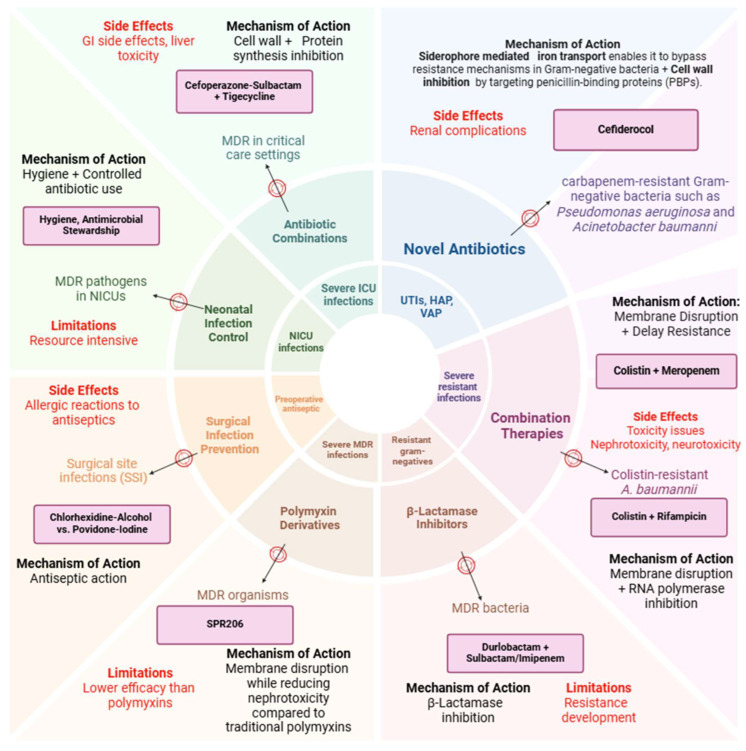
Overview of the antibiotics studied in this article (limitation, side effect, and mechanism of action).

1. Patient Population Heterogeneity: These studies included a diverse range of patients with varying comorbidities, ages, and baseline health conditions. This diversity can lead to significant variability in patient outcomes, as the effectiveness of treatments may differ greatly depending on individual patient characteristics such as renal function, immune status, and the presence of other underlying diseases. For instance, critically ill patients, those with pre-existing renal conditions, or those with multi-organ failure may experience different outcomes compared to healthier patients, which complicates the interpretation of treatment efficacy.

2. Limited Sample Sizes: Some studies, such as those on newer therapies like SPR206 or durlobactam, involved relatively small sample sizes, which reduces the generalizability of their findings. Small cohort sizes increase the risk of sampling bias and limit the ability to detect statistically significant differences in patient outcomes. Larger, multicenter trials are necessary to confirm these findings and assess the treatment’s effectiveness across broader populations.

3. Short-Term and Long-Term Efficacy: Many of the studies cited primarily focus on short-term outcomes, such as initial infection resolution and adverse events during the treatment period. However, long-term data on relapse rates, development of resistance, or sustained efficacy are often lacking. Variability in patient outcomes could be due to the lack of long-term follow-up, particularly for drugs like cefiderocol, which may show initial effectiveness but potentially contribute to complications or reinfection over time.

4. Differences in Treatment Regimens: The studies frequently involved variations in the dosing schedules and combination therapies, which can contribute to inconsistencies in patient outcomes. For example, patients who received cefiderocol alone may have had different results compared to those who were treated with cefiderocol in combination with other antibiotics. Additionally, variations in the dosing frequency, duration of therapy, and the presence of additional supportive treatments could have influenced the clinical outcomes observed.

5. Pathogen-Specific Variability: While some studies focus on specific pathogens like *Acinetobacter baumannii, Klebsiella pneumoniae*, and *Pseudomonas aeruginosa*, these bacteria exhibit significant genetic variability, which can impact how well they respond to treatment. For example, strains with different resistance profiles (e.g., carbapenemase-producing versus non-carbapenemase-producing) may not respond uniformly to the same antibiotic regimen. This could result in variability in treatment outcomes, with some patients experiencing better responses than others based on the pathogen’s resistance mechanisms.

6. Lack of Standardized Monitoring: Variability in patient outcomes can also stem from differences in how adverse events are monitored and reported. Some studies may not have rigorously tracked renal function or other key biomarkers, leading to inconsistent or underreported adverse events, such as nephrotoxicity. The absence of standardized monitoring protocols across different research centers could contribute to variations in the detection and management of treatment-related complications.

7. Differences in Clinical Settings: The clinical settings in which the studies were conducted (e.g., hospital-acquired infections versus community-acquired infections) may lead to variability in patient outcomes. For instance, patients in intensive care units (ICUs) are generally sicker and may have more complex infections, which could affect how well the treatments work and contribute to higher mortality rates. The outcomes observed in ICU settings may not be directly applicable to less critically ill populations.

8. Measurement of Outcomes: The way patient outcomes are measured can also introduce variability. Some studies rely on subjective clinical assessments, such as improvement in symptoms or physician evaluations of infection resolution, which can be influenced by the physician’s experience or interpretation. Others may use more objective measures, such as microbiological clearance or biomarkers, but even these can vary depending on the laboratory methods used across different centers. (Figure 3).

## 11. Conclusions

In conclusion, managing MDR infections remains a critical challenge despite recent advancements. Cefiderocol has proven effective against carbapenem-resistant bacteria, showing promise in treating hospital-acquired pneumonia and complicated urinary tract infections, though potential renal side effects require careful monitoring. Colistin, while still in use, shows limited benefits in combination therapies and raises concerns due to higher resistance-related mortality rates. New antibiotics like SPR206 and durlobactam offer hope for better treatments, and combining cefoperazone–sulbactam with tigecycline has shown superior results in tackling MDR *Acinetobacter baumannii* pneumonia. Additionally, infection prevention in surgical settings highlights the need for optimized treatment plans and improved protocols. While progress is evident, continuous research is essential to address evolving antibiotic resistance and improve outcomes for patients across diverse healthcare environments.

## Figures and Tables

**Table 1:** Comparative safety, efficacy, and adverse events of reviewed therapies.

Therapy	Efficacy Against Carbapenem-Resistant Bacteria	Primary Infections Treated	Common Adverse Events	Renal Toxicity	Liver Toxicity
Cefiderocol	High efficacy, particularly against metallo-β-lactamase-producing bacteria	HAP, complicated urinary tract infections	Diarrhea, fever, septic shock, vomiting, liver-related issues	High risk in critically ill patients	Increased liver enzymes
Colistin + Meropenem	Limited improvement in clinical outcomes	Carbapenem-resistant infections	Diarrhea, nausea, hypokalemia	Lower renal toxicity but still present	Rare liver toxicity
SPR206	Promising efficacy for MDR infections	Severe MDR infections	Mild paresthesia at higher doses	Minimal nephrotoxicity compared to polymyxins	No significant liver issues reported
Durlobactam + Sulbactam	Effective against β-lactamase-producing bacteria	Carbapenem-resistant *A. baumannii*	Mild transient liver effects	No significant renal toxicity	Mild liver effects
